# Current laboratory and clinical practices in reporting and interpreting anti-nuclear antibody indirect immunofluorescence (ANA IIF) patterns: results of an international survey

**DOI:** 10.1186/s13317-020-00139-9

**Published:** 2020-11-23

**Authors:** Lieve Van Hoovels, Sylvia Broeders, Edward K. L. Chan, Luis Andrade, Wilson de Melo Cruvinel, Jan Damoiseaux, Markku Viander, Manfred Herold, Wim Coucke, Ingmar Heijnen, Dimitrios Bogdanos, Jaime Calvo-Alén, Catharina Eriksson, Ana Kozmar, Liisa Kuhi, Carolien Bonroy, Bernard Lauwerys, Sofie Schouwers, Laurence Lutteri, Martine Vercammen, Miroslav Mayer, Dina Patel, William Egner, Kari Puolakka, Andrea Tesija-Kuna, Yehuda Shoenfeld, Maria José Rego de Sousa, Marcos Lopez Hoyos, Antonella Radice, Xavier Bossuyt

**Affiliations:** 1 Department of Laboratory Medicine, OLV Hospital, Aalst, Belgium; 2 Department of Microbiology and Immunology, KU Leuven, Leuven, Belgium; 3 Sciensano (Formerly Scientific Institute of Public Health), Brussels, Belgium; 4 Department of Oral Biology, University of Florida, Gainesville, FL USA; 5 Rheumatology Division, Universidade Federal de São Paulo, Escola Paulista de Medicina, Sao Paulo, Brazil; 6 Pontifícia Universidade católica de Goiás, Goiânia, Brazil; 7 Centraal Diagnostisch Laboratorium, MUMC, Maastricht, The Netherlands; 8 Department of Medical Microbiology and Immunology, University of Turku, Turku, Finland; 9 Rheumatology Laboratory, Department of Internal Medicine II, Medical University of Innsbruck, Innsbruck, Austria; 10 Medical Immunology, Laboratory Medicine, University Hospital Basel, Basel, Switzerland; 11 Department of Rheumatology and Clinical Immunology, Faculty of Medicine, School of Health Sciences, University of Thessaly, Larissa, Greece; 12 Servicio de Reumatología, Hospital Universitario Araba, Vitoria, Spain; 13 Department of Clinical Microbiology, Umeå University, Umeå, Sweden; 14 Department of Laboratory Diagnostics, University Hospital Centre Zagreb, Zagreb, Croatia; 15 Central Laboratory, East Tallin Central Hospital, Tallin, Estonia; 16 Department of Internal Medicine, Ghent University, Ghent, Belgium; 17 Department of Diagnostic Sciences, Ghent University, Ghent, Belgium; 18 Pôle de Pathologies Rhumatismales Et systémiques, Institut de Recherche Expérimentale et Clinique, Université Catholique de Louvain, Brussels, Belgium; 19 Department of Rheumatology, Cliniques Universitaires Saint-Luc, Brussels, Belgium; 20 Department of Laboratory Medicine, GZA Hospitals, Antwerp, Belgium; 21 Department of Clinical Chemistry, University Hospital Liège, Liège, Belgium; 22 Department of Laboratory Medicine, AZ Sint-Jan Hospital Bruges-Ostend, Bruges, Belgium; 23 Research Group Reproductive Immunology and Implantation (REIM), Vrije Universiteit Brussel, Brussels, Belgium; 24 Division of Clinical Immunology and Rheumatology, Department of Internal Medicine, University Hospital Centre Zagreb, Zagreb, Croatia; 25 UK NEQAS Immunology, Northern General Hospital, Immunochemistry & Allergy, Sheffield, UK; 26 Department of Medicine, South Karelia Central Hospital, Lappeenranta, Finland; 27 Laboratory of the Mosaic of Autoimmunity, Saint Petersburg State University, Saint-Petersburg, Russian Federation; 28 Zabludowicz Center for Autoimmune Diseases, Sheba Medical Center, Affiliated to Tel-Aviv University School of Medicine, Tel-Hashomer, Tel Aviv, Israel; 29 Centro de Medicina, Laboratorial Germano de Sousa, Lisboa, Portugal; 30 Servicio de Inmunología, Hospital Universitario Marqués de Valdecilla, Santander, Cantabria Spain; 31 UOC Microbiologia e Virologia, Presidio Ospedaliero San Carlo Borromeo, Milan, Italy; 32 Department of Laboratory Medicine, University Hospital Leuven, Leuven, Belgium

**Keywords:** Antinuclear antibodies, ANA patterns, Indirect immunofluorescence, ICAP

## Abstract

**Background:**

The International Consensus on Antinuclear Antibody (ANA) Patterns (ICAP) has recently proposed nomenclature in order to harmonize ANA indirect immunofluorescence (IIF) pattern reporting. ICAP distinguishes competent-level from expert-level patterns. A survey was organized to evaluate reporting, familiarity, and considered clinical value of ANA IIF patterns.

**Methods:**

Two surveys were distributed by European Autoimmunity Standardization Initiative (EASI) working groups, the International Consensus on ANA Patterns (ICAP) and UK NEQAS to laboratory professionals and clinicians.

**Results:**

438 laboratory professionals and 248 clinicians from 67 countries responded. Except for dense fine speckled (DFS), the nuclear competent patterns were reported by > 85% of the laboratories. Except for rods and rings, the cytoplasmic competent patterns were reported by > 72% of laboratories.

Cytoplasmic IIF staining was considered ANA positive by 55% of clinicians and 62% of laboratory professionals, with geographical and expertise-related differences.

Quantification of fluorescence intensity was considered clinically relevant for nuclear patterns, but less so for cytoplasmic and mitotic patterns. Combining IIF with specific extractable nuclear antigens (ENA)/dsDNA antibody testing was considered most informative.

Of the nuclear competent patterns, the centromere and homogeneous pattern obtained the highest scores for clinical relevance and the DFS pattern the lowest. Of the cytoplasmic patterns, the reticular/mitochondria-like pattern obtained the highest scores for clinical relevance and the polar/Golgi-like and rods and rings patterns the lowest.

**Conclusion:**

This survey confirms that the major nuclear and cytoplasmic ANA IIF patterns are considered clinically important. There is no unanimity on classifying DFS, rods and rings and polar/Golgi-like as a competent pattern and on reporting cytoplasmic patterns as ANA IIF positive.

## Keymessages

We report on an international survey on Antinuclear Antibody (ANA) indirect immunofluorescence (IIF) pattern reporting.

Both clinicians and laboratory professionals consider the major nuclear and cytoplasmic IIF patterns as clinically important.

There is no unanimity on reporting cytoplasmic patterns as ANA IIF positive.

## Background

Indirect immunofluorescence (IIF) on HEp-2 cells is commonly used to screen for antinuclear antibodies (ANA). ANA are helpful for the diagnosis of ANA-associated rheumatic diseases (AARD) [1, 2]. In the recent classification criteria for systemic lupus erythematosus, the presence of ANA is an entry criterion [3]. Besides, ANA are a prognostic marker for uveitis in children with juvenile idiopathic arthritis [4] and ANA positivity and titers are included in the internationally-accepted, simplified criteria for the diagnosis of autoimmune hepatitis [5–7]. Finally, some ANA patterns can point towards specific diseases such as the reticular/mitochondria-like pattern in primary biliary cholangitis and the centromere pattern in systemic sclerosis [8–10]. Guidelines for ANA detection by IIF recommend to report ANA titer and pattern [11, 12]. The higher the antibody level, the higher the likelihood for disease [13–15]. In addition, the combination of the antibody level and the antibody pattern can also provide helpful information [16].

The International Consensus on ANA Patterns (ICAP), a working group of the Autoantibody Standardization Committee (ASC) [17], has recently proposed an ordered nomenclature in order to harmonize the names and descriptions of distinct HEp-2 IIF pattern [18]. At the time of the survey (2019), 30 anti-cell (AC) patterns compose the ICAP classification tree [19]. The morphological description and examples of the AC patterns are accessible (in several languages) at the ICAP website (www.anapatterns.org and [20]). ICAP distinguishes patterns that should be readily recognized (competent-level) from patterns that require more experience (expert-level). This distinction is not only based on the fact that the pattern is easily recognizable, but also on its clinical relevance [17]. According to ICAP, the following AC patterns are competent-level: nuclear homogenous, speckled, dense fine speckled (DFS), centromere, nucleolar and discrete dots and cytoplasmic fibrillary, speckled, reticular/mitochondria-like, polar/Golgi-like and rods and rings. ICAP acknowledges that the distinction is a temporary status and is subject to feedback and commentaries from the international community [17].

In order to evaluate to which extent clinical laboratories and clinicians adhere to the ICAP recommendations and appreciate the clinical value of the IIF patterns, a survey on ANA IIF pattern reporting and interpretation was organized. The results of such surveys should guide further harmonization.

## Methods

The Belgian European Autoimmunity Standardization Initiative (EASI) working group prepared two structured survey forms, one addressed to laboratory professionals, reporting ANA IIF test results, and one to clinicians, requesting ANA IIF tests and interpreting the ANA IIF result report. Both survey forms (available as Additional file 1 and Additional file 2) interrogated reporting, familiarity and clinical significance of ANA IIF patterns. During a 2 year period (06/2017–05/2019), the survey forms were distributed to laboratory professionals and rheumatologists by the EASI working groups. Besides, the survey forms were also distributed through UK NEQAS and ICAP. The specialty of the clinicians that participated through ICAP is not known.

Globally, the surveys forms were distributed in the following formats:

1. open, web-based survey (Austria, Spain, Italy, Croatia, Sweden, UK, Switzerland, Portugal, Estonia, Greece)
2. closed survey (Belgium, Finland, the Netherlands, laboratories that are subscribed to ICAP with exclusion of the countries mentioned above as they were addressed through EASI)

Statistical analysis (chi-squared test relative proportion) was performed in MEDCALC (software version 17.1., Ostend, Belgium). A *p* value < 0.05 was considered as statistically significant.

## Results

### Characteristics of the participating laboratories and clinicians

Four hundred thirty-eight laboratory professionals and 248 clinicians (183 or 74% rheumatologists, for the other clinicians, specialty is not known) from 67 different countries worldwide responded to the surveys, of whom 358 (82%) and 84 (34%), respectively, completed the whole survey. Since the survey was also distributed through an open, web-based format we were unable to accurately estimate the response rate. Table 1 shows the geographic distribution of the respondents. Most of the respondents were from Europe (259/438 or 59% of laboratory professionals and 206/248 or 83% of the clinicians). Fifty percent (220/438) of the laboratory professionals that responded considered their laboratory as expert-level (i.e. recognize patterns that require more expertise) and 54% (135/248) of the clinicians worked in a tertiary hospital.

**Table 1:** Geographic distribution of the respondents with information on laboratories and clinicians showing (i) the geographic distribution of the respondents, (ii) the classification of laboratories as competent or expert and (iii) the clinical setting of the clinicians (N.a.: not applicable; Total completed: number of respondents that completed the whole survey)

Continent	Laboratory professionals				Clinicians				
	Competent	Expert	n.a	Total	Primary setting	Secundary setting	Tertiary setting	n.a	Total
Africa	6	4		10					
Asia	33	27	1	61		2	11	1	14
Australia	1	5		6			2		2
Europe	121	134	4	259	49	47	107	3	206
North-America	17	19	1	37	3	1	5	1	10
South-America	30	31	3	64	1	4	10		15
(Not assigned)	1			1	1				1
Total	209 (47.7%)	220 (50.2%)	9	438	54 (21.8%)	54 (21.8%)	135 (54.4%)	5	248
Total completed	166	186	6	358	11	11	60	2	84

Countries of origin of laboratory experts: Africa: Algeria, Egypt, Morocco, Saudi-Arabia, South-Africa, Tunisia; Asia: Azerbaijan, China, Hong Kong, India, Indonesia, Iran, Israel, Korea, Lebanon, Malaysia, Pakistan, Taiwan, Thailand, United Arab Emirates; Europe: Austria, Belgium, Bosnia and Herzegovina, Bulgaria, Croatia, Czech Republic, Denmark, Estonia, Finland, France, Germany, Greece, Hungary, Italy, Kazakhstan, Lithuania, Malta, Netherlands, Norway, Poland, Portugal, Romania, Serbia, Slovakia, Spain, Sweden, Switzerland, Turkey, United Kingdom; North-America: Canada, Guatemala, Mexico, Nicaragua, Panama, United States of America; South-America: Argentina, Bolivia, Brazil, Chile, Colombia, Costa Rica, Ecuador, Peru, Uruguay, Venezuela

Countries of origin of clinicians: Asia: China, India, Japan, Korea, Malaysia, Taiwan; Europe: Austria, Belgium, Croatia, Cyprus, Estonia, Finland, Greece, Hungary, Italy, Lithuania, Netherlands, Norway, Poland, Serbia, Slovakia, Spain, Sweden, Turkey, United Kingdom; North-America: Canada, Mexico, Dominican Republic, United States of America; South-America: Argentina, Brazil, Chile, Peru

### Reporting of ANA IIF patterns

Table 2 represents an overview of (i) the frequency of reporting of the various AC patterns, (ii) the estimation whether a pattern should be classified as competent, and (iii) the clinical relevance of the various AC patterns. For the clinical relevance score, 1 is the lowest and 5 the highest score.

**Table 2:** Overview of (i) the frequency of reporting of the various AC patterns, (ii) the estimation whether a pattern should be classified as competent, and (iii) the clinical relevance (1 is the lowest and 5 the highest score) of the various AC patterns. The reported range represents the minimum and maximum number of responders to the specific questions posed

		(i) Reporting (%)				(ii) Competent (%)			(iii) Clinical relevance score 1–2 (%)				(iii) Clinical relevance score 4–5 (%)			
		Laboratory			Clinicians	Laboratory			Laboratory			Clinicians	Laboratory			Clinicians
		Competent	Expert	Total		Competent	Expert	Total	Competent	Expert	Total		Competent	Expert	Total	
		n = 200–209	n = 213–220	n = 423–438	n = 170–177	n = 182–185	n = 202–208	n = 390–400	n = 175–182	n = 197–201	n = 378–388	n = 147–154	n = 175–182	n = 197–201	n = 378–388	n = 147–154
**Nuclear**																
**Homogeneous**	**AC-1**	**97.1**	**97.7**	**97.3**	**93.2**	**91.4**	**87.0**	**89.0**	**3.9**	**6.0**	**4.9**	**17.0**	**88.4**	**91.5**	**90.2**	**70.6**
**Speckled**	**AC-2,4,5**	**91.9**	**90.0**	**90.8**	**92.0**	**91.4**	**87.4**	**89.2**	**2.3**	**7.5**	**6.2**	**14.5**	**79.1**	**75.4**	**77.5**	**63.8**
**Dense fine speckled**	**AC-2**	**47.8**	**75.6**	**62.4**	**54.9**	**56.0**	**44.4**	**49.7**	**11.7**	**30.8**	**27.6**	**31.1**	**42.8**	**39.4**	**41.1**	**35.1**
Fine	AC-4	51.5	73.6	63.3	60.9	53.8	54.6	54.8	6.8	10.6	12.2	30.2	55.0	66.7	61.5	37.6
Large	AC-5	54.1	73.7	64.7	40.9	57.8	56.9	57.8	7.0	9.1	11.7	34.5	56.9	70.2	64.2	29.7
**Centromere**	**AC-3**	**99.0**	**98.2**	**98.4**	**90.3**	**89.7**	**87.3**	**88.4**	**0.8**	**4.5**	**3.1**	**7.8**	**93.4**	**93.0**	**93.3**	**83.8**
**Discrete nuclear dots**	**AC-6,7**	**85.4**	**84.9**	**85.2**	**72.4**	**87.0**	**80.8**	**83.8**	**4.1**	**8.0**	**8.3**	**29.1**	**65.2**	**63.8**	**64.5**	**43.7**
multiple	AC-6	60.8	85.4	73.8	43.6	65.8	48.8	57.1	8.1	10.6	13.5	44.6	47.8	68.2	58.3	25.0
few	AC-7	56.4	80.4	69.3	35.3	64.5	47.8	56.0	10.7	31.3	26.8	44.6	32.8	36.9	35.2	21.6
**Nucleolar**	**AC-8,9,10**	**94.7**	**94.0**	**94.2**	**83.9**	**90.8**	**86.3**	**88.4**	**3.4**	**6.0**	**6.5**	**17.4**	**77.2**	**80.4**	**79.2**	**61.1**
Homogeneous	AC-8	32.8	52.5	43.3	51.4	37.5	32.4	34.9	10.0	14.6	17.6	30.5	44.6	59.6	52.8	36.4
Clumpy	AC-9	20.8	46.0	34.0	18.6	25.7	20.6	23.4	11.3	15.7	19.4	47.6	38.4	60.1	50.1	23.1
Punctate	AC-10	17.8	46.0	32.9	22.8	24.0	19.6	22.1	12.6	15.7	20.8	46.3	35.2	60.6	48.9	21.1
Nuclear envelope	AC-11,12	68.1	85.3	77.0	34.3	75.4	69.6	72.3	6.3	12.1	12.5	47.6	54.2	68.7	61.9	23.8
Smooth	AC-11	19.7	49.1	35.3	19.4	26.2	20.1	23.6	13.4	23.2	25.4	51.0	29.2	47.0	38.7	17.0
Punctate	AC-12	17.8	43.1	31.4	17.1	20.8	13.2	17.5	12.8	19.2	22.8	51.0	30.3	57.6	45.0	17.7
Pleiomorphic	AC-13,14	49.5	70.7	60.6	28.1	59.2	49.5	54.4	10.9	16.1	19.3	49.0	36.9	52.8	45.3	22.4
PCNA	AC-13	46.8	83.4	65.7	45.9	39.9	32.0	36.1	9.9	14.1	17.3	32.2	44.4	60.1	53.1	38.9
CENP-F	AC-14	28.2	65.0	47.2	41.5	24.6	21.2	23.2	12.6	28.9	27.6	33.1	30.3	37.1	34.1	40.5
**Cytoplasmic**																
**Fibrillar**	**AC-15,16,17**	**67.8**	**76.4**	**72.1**	**50.3**	**72.3**	**70.1**	**71.4**	**11.3**	**23.7**	**23.6**	**43.9**	**39.5**	**41.4**	**41.2**	**28.4**
Linear	AC-15	48.0	72.6	61.0	35.1	50.3	40.7	45.2	11.5	15.7	19.7	47.3	44.1	56.6	50.9	22.3
Filamentous	AC-16	32.2	54.8	44.2	28.7	31.7	28.1	30.0	18.7	44.9	42.2	49.3	18.3	21.7	20.6	16.9
Segmental	AC-17	21.5	40.2	31.2	18.7	26.8	22.2	24.7	17.7	49.7	43.7	53.1	20.0	17.8	19.3	13.6
**Speckled**	**AC-18,19,20**	**67.8**	**81.3**	**74.8**	**52.0**	**72.8**	**73.5**	**73.2**	**7.3**	**19.7**	**17.5**	**47.0**	**50.0**	**50.0**	**50.3**	**28.2**
Discrete dots	AC-18	34.8	71.0	53.8	25.1	41.0	36.5	38.7	16.6	42.1	38.4	53.7	25.4	24.4	25.3	18.4
Dense fine speckled	AC-19	35.6	67.4	52.3	37.1	39.3	32.0	35.6	11.8	19.7	22.0	45.3	40.7	56.1	49.1	20.3
Fine speckled	AC-20	38.1	74.9	57.3	42.4	38.8	34.0	36.2	9.4	18.2	18.9	44.2	44.1	56.6	50.7	23.8
**Reticular**	**AC-21**	**65.0**	**89.9**	**78.1**	**49.4**	**72.1**	**75.0**	**73.8**	**5.5**	**6.6**	**8.9**	**39.6**	**72.3**	**84.8**	**79.3**	**38.9**
**Polar / Golgi-like**	**AC-22**	**71.4**	**87.6**	**79.5**	**42.1**	**67.2**	**63.5**	**65.3**	**17.6**	**43.9**	**40.4**	**53.4**	**33.9**	**21.7**	**27.3**	**23.6**
**Rods and rings**	**AC-23**	**52.5**	**72.8**	**62.7**	**27.1**	**60.7**	**53.7**	**56.9**	**20.8**	**52.3**	**47.9**	**51.7**	**24.3**	**18.8**	**21.3**	**23.8**
Mitotic																
Centrosome	AC-24	61.1	78.6	70.3	43.5	57.1	48.5	52.8	18.4	54.3	46.6	43.9	23.2	20.3	21.6	30.4
Spindle fibers	AC-25	61.6	82.7	72.5	31.8	57.9	46.6	52.2	18.3	51.5	45.0	48.6	23.6	19.7	21.5	20.3
NuMA	AC-26	44.8	75.9	61.0	31.0	44.0	32.0	38.1	16.0	37.9	35.7	52.3	26.0	32.3	29.7	24.2
Intercellular bridge	AC-27	45.3	74.4	60.2	24.1	48.4	39.4	43.7	20.5	56.9	50.0	54.4	19.8	17.3	18.7	13.6
Mitotic chromosomal	AC-28	24.8	47.9	36.8	24.1	27.5	24.1	25.8	21.6	57.4	51.3	56.5	16.4	13.2	15.0	15.0

For each question, the results per pattern, are presented as percentages of the total laboratory (competent and expert reported separately) and clinician respondents. The competent patterns are highlighted in bold. The reported range represents the minimum and maximum number of responders to the specific questions posed

For each question, the results per pattern, are presented as percentages of the total laboratory (competent and expert reported separately) and clinician respondents. The competent patterns are highlighted in bold. The reported range represents the minimum and maximum number of responders to the specific questions posed.

The competent-level patterns centromere (AC-3), homogeneous (AC-1), speckled (AC-2,4,5) and nucleolar (AC-8,9,10) patterns were used in ANA IIF test result reporting by > 90% of competent-level and expert-level laboratories (Table 2). The nuclear dot patterns (AC-6,7) were reported by 85% of the laboratories and the DFS pattern (AC-2) by 62% of the laboratories. Clinicians reported comparable (or somewhat lower) frequencies of being acquainted with those patterns (Table 2).

Cytoplasmic AC patterns were less frequently used in ANA IIF test result reporting than nuclear AC patterns. Among the competent cytoplasmic patterns (fibrillar (AC-15,16,17), speckled (AC-18,19,20), reticular/ mitochondria-like (AC-21), polar/Golgi-like (AC-22), rods and rings (AC-23), the rods and rings pattern (AC-23) was the pattern that was least used (63% of the laboratories) (Table 2).

The competent-level DFS pattern (AC-2) and rods and rings pattern (AC-23) were significantly (p < 0.0001) more used by expert-level laboratories than by competent-level laboratories (76% versus 48% for DFS and 73% versus 53% for rods and rings). Moreover, the DFS pattern was less reported by laboratories in North-America (44%) and Europe (57%) than by laboratories in the other continents (Table 3).

**Table 3:** Demographic differences in reporting the dense fine speckled (AC-2) and the rods and rings pattern (AC-23), with results shown and presented as percentage (%) of the total responders % per geographic continent

a) Dense fine speckled (AC-2)								
	Laboratory professionals						Clinicians	
	Total		Competent		Expert			
Continent	n	Reporting (%)	n	Reporting (%)	n	Reporting (%)	n	Reporting (%)
Africa	8	6 (75.0%)^a^	5	3 (60.0%)	3	3 (100%)	0	n.a
Asia	59	46 (78.0%)^b^	33	22 (66.7%)	26	24 (92.3%)	9	5 (55.6%)
Australia	6	5 (83.3%)^c^	1	0 (0.0%)	5	5 (100%)	2	1 (50.0%)
Europe	251	143 (57.0%)	118	48 (40.7%)	133	95 (71.4%)	143	78 (54.5%)
North Amercia	36	16 (44.4%)	17	7 (41.2%)	19	9 (47.4%)	7	2 (28.6%)
South America	61	46 (75.4%)^d^	30	18 (60.0%)	31	28 (90.3%)	11	8 (72.7%)
Total	421	262 (62.2**%**)	204	98 (48.0**%**)	217	164 (75.6**%**)	172	94 (54.7%)

b) Rods and rings (AC-23)								
	Laboratory						Clinicians	
	Total		Competent		Expert			
Continent	n	Reporting (%)	n	Reporting (%)	n	Reporting (%)	n	Reporting (%)
Africa	10	8 (80.0%)	6	5 (83.3%)	4	3 (75.0%)	0	n.a
Asia	59	45 (76.3%)	33	23 (69.7%)	26	22 (84.6%)	9	3 (33.3%)
Australia	5	1 (20.0%)	1	0 (0.0%)	4	1 (25.0%)	2	0 (0.0%)
Europe	247	149 (60.3%)	116	55 (47.4%)	131	94 (71.8%)	140	33 (23.6%)
North Amercia	36	22 (61.1%)	17	8 (47.1%)	19	14 (73.7%)	7	2 (28.6%)
South America	59	36 (61.0%)	30	15 (50.0%)	29	21 (72.4%)	11	3 (27.3%)
Total	416	261 (62.7%)	203	106 (52.2%)	213	155 (72.8%)	169	41 (24.3%)

^a^*p* = 0.1217 versus North-America and *p* = 0.3115 versus Europe

^b^*p* = 0.0009 versus North-America and *p* = 0.0030 versus Europe

^c^*p* = 0.0813 versus North-America and *p* = 0.1985 versus Europe

^d^*p* = 0.0022 versus North-America and *p* = 0.0084 versus Europe

*N.a.* not applicable

### Distinction between competent and expert patterns

Laboratory professionals were also interrogated whether they would classify a pattern as competent or expert-level. For most of the nuclear patterns there was a good agreement (84–89%) between the ICAP classification and the provided responses, except for the DFS pattern. Only 50% of the respondents would classify this pattern as competent (Table 2). Of interest, 72% of the respondents considered the nuclear envelope pattern a competent pattern rather than an expert pattern.

For the cytoplasmic patterns considered competent-level by ICAP, 71%—74% of the respondents consider the fibrillary, speckled and reticular/mitochondria-like pattern a competent pattern, 65% considered the polar/Golgi-like pattern a competent pattern and 57% considered the rods and rings pattern a competent pattern (Table 2).

### Are cytoplasmic patterns considered ANA positive?

Sixty-one percent of the clinicians (n = 105) and 54% of the laboratory professionals (n = 346) considered cytoplasmic HEp-2 cell IIF staining as ANA IIF positive. There were more expert-level laboratory professionals (61%) than competent-level laboratory professionals (46%) that considered cytoplasmic patterns as ANA positive (p = 0.0062). The fraction of laboratory professionals that considered cytoplasmic ANA patterns as ANA positive was higher in non-European countries (63%) than in European countries (48%) (p = 0.0075) (Table 4a). However, within Europe, differences between countries were observed. In Austria (15%; n = 2/13), the Netherlands (12%; n = 3/25), Sweden (13%; n = 1/8), Switzerland (29%; n = 2/7), Turkey (20%; n = 1/5) and the United Kingdom (22%; n = 2/9), cytoplasmic ANA IIF patterns were considered ANA positive by < 30% of laboratory professionals, while in Belgium (65%; n = 22/34), France (57%; n = 4/7), Italy (61%; n = 11/18), Portugal (78%; n = 18/23) and Spain (67%; n = 14/21), cytoplasmic patterns are considered ANA positive by > 60% of the laboratory professionals (Table 4b).

**Table 4:** Geographic variation in considering cytoplasmic ANA IIF patterns as ANA positive

a) The results are presented as absolute numbers and in percentage (%) of the responders per geographic continent						
	Total		Competent		Expert	
Continent	n	Reporting (%)	n	Reporting (%)	n	Reporting (%)
Africa	5	3 (60.0%)	4	2 (50.0%)	1	1 (100.0%)
Asia	48	32 (66.7%)	26	16 (61.5%)	22	16 (72.7%)
Australia	5	0 (0.0%)	1	0 (0.0%)	4	0 (0.0%)
Europe	211	102 (48.3%)	98	40 (40.8%)	113	62 (54.9%)
North Amercia	28	18 (64.3%)	12	6 (50.0%)	16	12 (75%)
South America	49	32 (65.3%)	23	12 (52.2%)	26	20 (76.9%)
Total	346^a^	187 (54.1%)	164	76 (46.3**%**)	182	111 (61.0**%**)

b) The results are presented as percentage (%) of the total responders per participating European country						
	Total		Competent		Expert	
Country	n	ANA positive (%)	n	ANA positive (%)	n	ANA positive (%)
Austria	13	2 (15.4%)	3	0 (0.0%)	10	2 (20.0%)
Belgium	34	22 (64.7%)	25	13 (52%)	9	9 (100.0%)
Bosnia and Herzegovina	1	1 (100.0%)	1	1 (100.0%)	n.a	n.a
Bulgaria	1	0 (0.0%)	n.a	n.a	1	0 (0.0%)
Croatia	6	3 (50.0%)	2	1 (50.0%)	4	2 (50.0%)
Czech Republic	1	0 (0.0%)	1	0 (0.0%)	n.a	n.a
Denmark	1	0 (0.0%)	1	0 (0.0%)	n.a	n.a
Estonia	5	5 (100.0%)	2	2 (100.0%)	3	3 (100.0%)
Finland	4	2 (50.0%)	2	1 (50.0%)	2	1 (50.0%)
France	7	4 (57.1%)	4	2 (50.0%)	3	2 (66.7%)
Germany	1	0 (0.0%)	n.a	n.a	1	0 (0.0%)
Greece	7	3 (42.9%)	2	1 (50.0%)	5	2 (40.0%)
Hungary	3	0 (0.0%)	n.a	n.a	3	0 (0.0%)
Italy	18	11 (61.1%)	5	2 (40.0%)	13	9 (69.2%)
Kazakhstan	2	2 (100.0%)	2	2 (100.0%)	n.a	n.a
Malta	1	1 (100.0%)	n.a	n.a	1	1 (100%)
Netherlands	25	3 (12.0%)	20	2 (10.0%)	5	1 (20.0%)
Norway	1	0 (0.0%)	n.a	n.a	1	0 (0.0%)
Poland	3	2 (66.7%)	n.a	n.a	3	2 (66.7%)
Portugal	23	18 (78.3%)	8	6 (75.0%)	15	12 (80.0%)
Romania	2	2 (100.0%)	n.a	n.a	2	2 (100.0%)
Serbia	1	0 (0.0%)	1	0 (0.0%)	n.a	n.a
Slovakia	1	1 (100.0%)	n.a	n.a	1	1 (100.0%)
Spain	21	14 (66.7%)	8	5 (62.5%)	13	9 (69.2%)
Sweden	8	1 (12.5%)	1	0 (0.0%)	7	1 (14.3%)
Switzerland	7	2 (28.6%)	n.a	n.a	7	2 (28.6%)
Turkey	5	1 (20.0%)	2	1 (50.0%)	3	0 (0.0%)
United Kingdom	9	2 (22.2%)	9	2 (22.2%)	0	0 (0.0%)
Total	211	102 (48.3%)	99	41 (41.4%)	112	61 (54.5%)

^a^n = 6 laboratories reported expertise level ‘not applicable’ and n = 1 no country

### Sub-specification of patterns

Less than half of the clinicians and laboratory professionals found it important to sub-specify (i) nucleolar patterns into homogeneous, clumpy and speckled (AC-8,9,10) (respectively 45% and 34%), (ii) cytoplasmic fibrillary patterns into linear, filamentous and segmental (AC-15,16,17) (respectively 28% and 42%) and (iii) cytoplasmic speckled patterns into discrete dots, dense fine speckled and fine speckled (AC-18,19,20) (respectively 42% and 53%). Seventy-one percent of the clinicians and 86% of the laboratory professionals found it important to report the reticular/mitochondria-like cytoplasmic pattern (AC-21). Sixty-two percent of the laboratory professionals distinguished multiple nuclear dots from few nuclear dots (AC-6,7) (data not shown).

The most used sub-specifications in ANA IIF result reports are nuclear fine speckled AC-4 (63%), nuclear coarse speckled AC-5 (65%), multiple nuclear dots AC-6 (74%) and few nuclear dots AC-7 (69%). These sub-specifications are significantly more reported by expert-level than by competent-level laboratories (*p* < 0.0001 for all sub-patterns). However, clinicians are not aware of such ANA IIF sub-specification and do not consider them clinically relevant (Table 2).

### Confirmation of IIF by specific tests

There was no difference between competent-level and expert-level laboratories regarding follow-up testing for antibodies to extractable nuclear antigens (ENA) and to dsDNA in case of a positive ANA IIF test. A geographical difference, however, was observed (Additional file 3. Table S5). Forty-one percent of European laboratories, but only 13% of North American and 10% of South American laboratories, systematically performed follow-up testing for anti-ENA antibodies and to dsDNA in case of a positive ANA IIF test or on clinical indication when ANA IIF is negative. Forty-seven percent of the North-American laboratories and 44% of the South-American ones, but only 16% of the European laboratories only performed confirmation testing when the tests were specifically requested (Additional file 3. Table S5).

The majority of competent (72%; n = 165) and expert laboratories (79%; n = 179) review their ANA IIF results after ENA/dsDNA confirmation testing, but only a minority would change the results (respectively 8% and 10%) or add a comment if clinically relevant (respectively 36 and 46%).

### Interpretation and clinical significance of ANA IIF patterns

Both clinicians (82%; n = 246) and laboratory professionals (81%; n = 352) considered the combination of an ANA IIF test result with a corresponding specific anti-ENA or anti-dsDNA test result as the most clinically relevant information.

The quantification of the nuclear ANA IIF fluorescence intensity was found clinically relevant by 79% of laboratory professionals and by 74% of clinicians (Additional file 4. Table S6). Thirty-three percent of laboratory professionals and 22% of clinicians reported that one should not titrate cytoplasmic patterns. Forty-three percent of laboratory professionals and 33% of clinicians reported that mitotic patterns should not be titrated (Additional file 4. Table S6).

Fifty-four percent of the clinicians (n = 107) reported that they would take medical decisions based on ANA IIF titer, whereas 87% of the clinicians reported that they would take medical decision based on results of specific testing for anti-ENA or anti-dsDNA.

Clinicians and laboratory professionals were also interrogated on how they appraise the clinical relevance of the various ANA patterns. Overall, clinicians scored the clinical relevance of the various patterns lower than laboratory professionals (Table 2). The highest scores for clinical relevance were obtained for the centromere (AC-3) pattern and the homogeneous (AC-1) pattern. Ninety-three percent of the laboratory professionals and 84% of the clinicians scored the clinical relevance of the centromere pattern high, and 90.2% of the laboratory professionals and 70.6% of the clinicians scored the clinical relevance of the homogeneous pattern high. Seventy-seven to 80% of the laboratory professionals and 61–64% of the clinicians scored the clinical relevance of the nuclear speckled pattern (AC-2,4,5) and nucleolar pattern (AC-8,9,10) high. In contrast, only 41% of laboratory professionals and 35% of clinicians scored the clinical relevance of the DFS pattern (AC-2) high.

Of the cytoplasmic patterns, the reticular/mitochondria-like pattern (AC-21) obtained the highest score for clinical relevance by laboratory professionals. The other competent cytoplasmic patterns received lower scores, with the lowest scores for polar/Golgi-like (AC-22) and rods and rings (AC-23).

The mitotic patterns obtained low scores for clinical relevance overall with ≤ 30% of the clinicians and laboratory professionals scoring these patterns as clinically relevant.

## Discussion

In the current paper, we present the results of a recent international survey on the reporting and interpretation of ANA IIF results in current daily practice. The results revealed concordances but also discordances with the recommendations posed by ICAP [18].

The survey confirmed that there is no consensus on whether anti-cytoplasmic antibodies should be considered ANA IIF positive or negative [21, 22]. Even within Europe, there are divergences between countries, as the geographic block represented by Portugal, Spain, Italy, France and Belgium tend to report exclusive cytoplasmic reactivity as a positive test, whereas countries out of this axis only report nuclear reactivity as a positive test. ANA is the common clinical and laboratory term used for more than 50 years. The term is maintained for historical reasons as well as for laboratory coding and invoicing. However, the name ‘antinuclear’ for the HEp-2 cell IIF test does not take into consideration that autoantibodies to cell compartments other than the nucleus (i.e. cytoplasm, mitotic) are also detected and that these antibodies may be clinically relevant [10]. Therefore, the ICAP executive board, already in their first report, advocates that in situations where there is a clear cytoplasmic or mitotic apparatus reactivity, these results need to be reported to the clinician. However, even within ICAP, the discussion on whether those patterns should be regarded as ANA IIF positive or negative has not reached consensus [10, 17, 23]. Being aware that the term ‘Antinuclear antibody (ANA) test’ is inappropriate, the use of the suggested alternative name, HEp-2 IIF test [10], will foster harmonization of reporting ANA IIF results.

The familiarity with ANA IIF patterns is mainly clear for the nuclear competent patterns (with the exception of DFS) and the cytoplasmic reticular/mitochondria-like antibody (AC-21) pattern, findings which are in concordance to the results of a similar recent survey performed by the American association of Medical Laboratory Immunologists [24]. Clinicians were less familiar with the cytoplasmic reticular pattern than laboratory professionals, which might be related to the fact that the survey was mainly directed to rheumatologists (and not hepato-gastroenterologists). Overall, cytoplasmic and mitotic patterns are less reported by laboratories than nuclear patterns. The integration of computer-aided immunofluorescence microscopy could further improve the familiarity and consistency in pattern assignments [25, 26], although currently the existing systems are able to recognize only a small fraction of patterns.

The survey shows that not all laboratories report the DFS pattern and the rods and ring pattern and that not all expert-level laboratory professionals are convinced that these two patterns should be classified as competent.

The DFS pattern (AC-2) can vary depending on the manufacturer of the HEp-2 cell substrates [27, 28] and its correct identification can be challenging, resulting in a high frequency of cases misclassified as DFS pattern [28, 29]. Furthermore, the assumed clinical relevance of the DFS pattern to exclude AARD only holds if the specificity is confirmed and if it is monospecific for DFS70 [10, 27, 30]. The inclusion of anti-DFS70 antibody testing in a diagnostic algorithm or reflex testing is currently under discussion [31, 32]. It has been proposed to better define the DFS AC-2 pattern in the ICAP classification algorithm including a new pattern called ‘pseudo-DFS’ referring to the nuclear speckled pattern with clear staining of the metaphase plate, but without the typical features of the DFS (AC-2) pattern (i.e. a more homogeneous distribution and more uniform brightness of the nuclear speckles than in the typical DFS pattern). Distinguishing this pattern from the classical DFS pattern would be important to increase the accuracy in the interpretation of the ANA IIF test [33]. The recent availability of a reference serum with mono-reactivity to DFS70 by the Autoantibody Standardization Committee of the International Union of Immunology Societies (www.autoab.org) should help laboratories across the world to improve the ability in recognizing the DFS (AC-2) pattern [34].

In contrast to the DFS pattern, the rods and rings (AC-23) pattern is easily recognizable and there is a defined clinical association (Hepatitis C virus infection under treatment with α-interferon and ribavirin) [10]. However, as mentioned in the ICAP nomenclature tree (www.anapatterns.org), the AC-23 ANA IIF pattern is only detectable in selected HEp-2 cell slides. Thus, depending on the slide source (i.e. manufacturer) used, labs will be able or unable to report this pattern. This likely explains in part the relatively low recognition rate of this pattern even in expert laboratories.

Overall, the patterns that are most frequently reported are the patterns that are considered to have the highest clinical relevance, with some exceptions such as the centrosome, Golgi and spindle fiber patterns, which are reported by circa 80% of the laboratories despite not having peculiar clinical associations. In addition, the DFS pattern is reported in less than 50% of laboratories. Considering that this pattern includes the staining of the metaphase plate, the DFS pattern can be misclassified as nuclear homogeneous pattern (AC-1), which has completely different immunological and clinical implications.

In daily routine practice, sub-specifying nucleolar (AC-8,9,10) patterns, cytoplasmic fibrillar patterns and cytoplasmic speckled patterns is infrequently performed, even among experts. However, clinicians do not consider such sub-classification clinically relevant (Table 2).

The highest scores for clinical relevance were obtained for the centromere (AC-3) and homogeneous (AC-1) pattern, followed by the nuclear speckled (AC-2,4,5) and cytoplasmic reticular (AC-21) patterns. Especially the centromere pattern is associated with a specific disease (limited systemic sclerosis, primary biliary cholangitis and Sjögren’s syndrome) [16]. It is the only pattern that has an almost absolute association with a restrict set of autoantibody specificities (anti-CENP-B and/or anti-CENP-A). For all other patterns, follow-up testing to identify the target antigen is mandatory, and the pattern can help to guide such follow-up testing [10].

The clinical significance of the cytoplasmic patterns as a whole was considered lower compared to nuclear patterns. Even cytoplasmic patterns such as the (dense) fine speckled patterns that are associated with clinically relevant antibodies such as the anti-synthetase antibodies, anti-SRP and anti-Rib-P are not widely recognized as clinically relevant patterns. This perception clearly indicates the need for implementing education programs in order to increase the awareness of such associations and improve the interpretation and optimal use of the ANA IIF test. The polar/Golgi-like pattern is considered a competent pattern. It is easily recognizable but its clinical association with a disease is limited [10, 35–37].

The quantification of the ANA IIF fluorescence intensity was found clinically relevant for the nuclear ANA IIF patterns by a substantial fraction of clinicians (74%) and laboratory experts (79%). The importance of the autoantibody titer (level) is recognized by guidelines and recommendations [1, 3, 11, 12]. Recent studies showed that the likelihood for AARD increases with increasing ANA IIF fluorescence intensity [13–15]. However, important variation in the detection and titration of ANAs has been reported with manual and automated methods [38–41].

Clinicians are aware of the differences between laboratories for both ANA IIF and specific ENA/dsDNA testing. A low percentage of clinicians take medical decisions based on the ANA IIF titer (54%).

Clinicians and laboratory professionals considered that combined information on HEp-2 ANA and specific tests for anti-ENA and anti-dsDNA antibodies is most informative. This is in line with recent studies that showed that combining an immunoassay with ANA IIF adds value if the results of both tests are correctly judged in the context of the clinical manifestations of the patient [42, 43]. Furthermore, combining IIF with a solid-phase assay can assist in patient stratification, especially in case of a low-positive ANA IIF titer [42, 44, 45]. ICAP promotes to integrate the ANA IIF titer and pattern with advice for follow-up testing, taking into account the clinical presentation of the patient [10, 46].

## Conclusions

In conclusion, this international survey confirms that the major nuclear (homogenous, speckled, centromere, nucleolar and dots) and cytoplasmic (reticular) patterns are considered clinically important. It also supports the ICAP classification in competent and expert patterns for the majority of the competent patterns, but there is no unanimity on classifying DFS, rods and rings and polar/Golgi-like as a competent pattern. Compared to nuclear patterns, cytoplasmic patterns are less reported and their clinical relevance is considered lower than the clinical relevance of nuclear patterns. Combining IIF with testing for specific antibodies is considered to be most informative. There is no unanimity among the respondents regarding the question whether a cytoplasmic pattern is ANA IIF positive.

## Supplementary information

**Additional file 1.** International EASI questionnaire ANA IIF patterns for Clinicians.

**Additional file 2.** International EASI questionnaire ANA IIF patterns for laboratory professionals.

**Additional file 3.** **Table S5.** Demographic differences in confirming ANA IIF results with specific tests for anti-ENA and anti-dsDNA.

**Additional file 4.** **Table S6.** Overview of the fraction of respondents (laboratory professionals or clinicians) that considered it important to report the fluorescence intensity of nuclear, cytoplasmic or mitotic ANA IIF patterns.

## Data Availability

The datasets used and analyzed during the current study are available from the corresponding author on reasonable request.

## Abbreviations

AARD: Antinuclear antibody associated rheumatic diseases
AC: Anti-cell
ANA: Antinuclear antibody
ASC: Autoantibody Standardization Committee
ICAP: The International Consensus on Antinuclear Antibody Patterns
IIF: Indirect immunofluorescence
EASI: European Autoimmunity Standardization Initiative
DFS: Dense fine speckled
ENA: Extractable nuclear antigens
