# Supplementary material for: Current laboratory and clinical practices in reporting and interpreting anti-nuclear antibody indirect immunofluorescence (ANA IIF) patterns: results of an international survey
Source: Auto Immun Highlights. 2020 Nov 23;11(1):17. doi: 10.1186/s13317-020-00139-9 (PMC7684889; doi:10.1186/s13317-020-00139-9)
Supplement: Supplementary file 1 — Additional file 1. International EASI questionnaire ANA IIF patterns for Clinicians. [file 13317_2020_139_MOESM1_ESM.docx]

**Supplemental data International EASI questionnaire ANA IIF patterns for Clinicians.**

**Accompanying letter**

Dear colleague,

We invite you to participate in a survey on antinuclear antibody (ANA) testing organized by the Belgian branch of the European Autoimmunity Standardization Initiative (EASI) and supported by ICAP.

EASI was founded in 2001 with the intention of improving diagnostics in chronic rheumatic disorders by strengthening the collaboration between clinical and laboratory scientists responsible for autoimmune diagnostics in Europe. One of the aims of EASI is to harmonize algorithms for cost-effective and rational autoantibody testing and the international conceptualization of standardization in this area. In this context, the recent International Consensus on ANA Patterns (ICAP) initiative, in which a standardized way to report ANA patterns is proposed, means an important step in this direction. To extend this initiative and gather worldwide information on ANA pattern recognition and the clinical relevance of specific ANA patterns, we’d like to ask for your cooperation in completing the survey. Two different surveys have been established: one for laboratory professionals performing ANA tests and another for clinicians.

The survey contains 22 questions and takes about 15 minutes to complete. It is possible to save the survey after each page of questions and to continue later. Hereto, click RESUME LATER. The survey will be open till 31/03/2019. Please have a look at the ICAP website (www.anapatterns.org) before filling out the questionnaire. Your information will be handled confidentially and processed anonymously.

We thank you in advance for your participation.

Sincerely,

In name of the Belgian EASI working group: Apr. Lieve Van Hoovels, Apr. Sofie Schouwers, Prof. Xavier Bossuyt, Dr. Sylvia Broeders;

And of the International Consensus on ANA patters (ICAP) organizers: Dr. Edward Chan, Dr. Luis Andrade

There are 22 questions in this survey

**1. Setting clinical practice**

1.1 In which country are you active?

Please choose only one of the following: (< List of countries in alphabetic order)

1.2 In which type of organization are you working?

Please choose only one of the following:

- Academic institution
- Clinical/Diagnostic Laboratory
- Hospital/Clinic
- Industry/Business
- Other

1.3 Which date did you consult http://www.anapatterns.org/?

Please enter a date:

1.4 Do you perform medicine in:

Please choose only one of the following:

- primary setting
- secundary setting
- tertiary setting
- not applicable

1.5 Do you consider the laboratory you work with as an expert-level or

competent-level for ANA IIF analysis?

Please choose only one of the following:

- expert-level
- competent-level
- not applicable

**2. ANA IIF analysis**

2.1 What do you consider clinically more important:

Please choose only one of the following:

- a specific ANA IIF pattern
- the identification of a specific ENA/dsDNA antibody
- a corresponding ANA IIF pattern and specific ENA/dsDNA antibody identification
- not applicable

**3. ANA IIF reporting (1)**

3.1 Are you familiar with the mentioned pattern?

Please choose the appropriate response for each item:

|  | Yes | No |
| --- | --- | --- |
| Nuclear: Homogeneous (AC-1)  Nuclear: Speckled (AC-2,4,5)  Nuclear: Dense fine speckled (AC-2)  Nuclear: Fine Speckled (AC-4)  Nuclear: Large/Coarse speckled (AC-5)  Nuclear: Centromere (AC-3)  Nuclear: Nuclear dots (AC-6,7)  Nuclear: Multiple nuclear dots (AC-6)  Nuclear: Few nuclear dots (AC-7)  Nuclear: Nucleolar (AC-8,9,10)  Nuclear: Homogeneous nucleolar (AC-8)  Nuclear: Clumpy nucleolar (AC-9)  Nuclear: Punctate nucleolar (AC-10)  Nuclear: Nuclear envelope (AC-11,12)  Nuclear: Smooth nuclear envelope (AC-11)  Nuclear: Punctate nuclear envelope (AC-12)  Nuclear: Pleomorphic (AC-13,14)  Nuclear: PCNA-like (AC-13)  Nuclear: CENP-F-like (AC-14) | o  o  o  o  o  o  o  o  o  o  o  o  o  o  o  o  o  o  o | o  o  o  o  o  o  o  o  o  o  o  o  o  o  o  o  o  o  o |
| Cytoplasmic: Fibrillar (AC-15,16,17)  Cytoplasmic: Linear/actin (AC-15)  Cytoplasmic: Filamentous/microtubules (AC-16)  Cytoplasmic: Segmental (AC-17)  Cytoplasmic: Speckled (AC-18,19,20)  Cytoplasmic: Discrete dots (AC-18)  Cytoplasmic: Dense Fine Speckled (AC-19)  Cytoplasmic: Fine speckled (AC-20)  Cytoplasmic: Reticular/AMA (AC-21)  Cytoplasmic: Polar/Golgi like (AC-22)  Cytoplasmic: Rods and rings (AC-23) | o  o  o  o  o  o  o  o  o  o  o | o  o  o  o  o  o  o  o  o  o  o |
| Mitotic: Centrosome (AC-24)  Mitotic: Spindle fibers (AC-25)  Mitotic: NuMa-Like (AC-26)  Mitotic: Intercellular bridge (AC-27)  Mitotic: Mitotic chromosomal envelope (AC-28) | o  o  o  o  o | o  o  o  o  o |

3.2 Are the following patterns reported by your laboratory

Please choose the appropriate response for each item:

|  | Yes | No |
| --- | --- | --- |
| Nuclear: Homogeneous (AC-1)  Nuclear: Speckled (AC-2,4,5)  Nuclear: Dense fine speckled (AC-2)  Nuclear: Fine Speckled (AC-4)  Nuclear: Large/Coarse speckled (AC-5)  Nuclear: Centromere (AC-3)  Nuclear: Nuclear dots (AC-6,7)  Nuclear: Multiple nuclear dots (AC-6)  Nuclear: Few nuclear dots (AC-7)  Nuclear: Nucleolar (AC-8,9,10)  Nuclear: Homogeneous nucleolar (AC-8)  Nuclear: Clumpy nucleolar (AC-9)  Nuclear: Punctate nucleolar (AC-10)  Nuclear: Nuclear envelope (AC-11,12)  Nuclear: Smooth nuclear envelope (AC-11)  Nuclear: Punctate nuclear envelope (AC-12)  Nuclear: Pleomorphic (AC-13,14)  Nuclear: PCNA-like (AC-13)  Nuclear: CENP-F-like (AC-14) | o  o  o  o  o  o  o  o  o  o  o  o  o  o  o  o  o  o  o | o  o  o  o  o  o  o  o  o  o  o  o  o  o  o  o  o  o  o |
| Cytoplasmic: Fibrillar (AC-15,16,17)  Cytoplasmic: Linear/actin (AC-15)  Cytoplasmic: Filamentous/microtubules (AC-16)  Cytoplasmic: Segmental (AC-17)  Cytoplasmic: Speckled (AC-18,19,20)  Cytoplasmic: Discrete dots (AC-18)  Cytoplasmic: Dense Fine Speckled (AC-19)  Cytoplasmic: Fine speckled (AC-20)  Cytoplasmic: Reticular/AMA (AC-21)  Cytoplasmic: Polar/Golgi like (AC-22)  Cytoplasmic: Rods and rings (AC-23) | o  o  o  o  o  o  o  o  o  o  o | o  o  o  o  o  o  o  o  o  o  o |
| Mitotic: Centrosome (AC-24)  Mitotic: Spindle fibers (AC-25)  Mitotic: NuMa-Like (AC-26)  Mitotic: Intercellular bridge (AC-27)  Mitotic: Mitotic chromosomal envelope (AC-28) | o  o  o  o  o | o  o  o  o  o |

**4. ANA IIF reporting (2)**

4.1 Indicate the patterns you consider clinically relevant and give an estimate of

the importance: 1 (clinically not important) and 5 (clinically very important)

Please choose the appropriate response for each item:

|  | 1 | 2 | 3 | 4 | 5 |
| --- | --- | --- | --- | --- | --- |
| Nuclear: Homogeneous (AC-1)  Nuclear: Speckled (AC-2,4,5)  Nuclear: Dense fine speckled (AC-2)  Nuclear: Fine Speckled (AC-4)  Nuclear: Large/Coarse speckled (AC-5)  Nuclear: Centromere (AC-3)  Nuclear: Nuclear dots (AC-6,7)  Nuclear: Multiple nuclear dots (AC-6)  Nuclear: Few nuclear dots (AC-7)  Nuclear: Nucleolar (AC-8,9,10)  Nuclear: Homogeneous nucleolar (AC-8)  Nuclear: Clumpy nucleolar (AC-9)  Nuclear: Punctate nucleolar (AC-10)  Nuclear: Nuclear envelope (AC-11,12)  Nuclear: Smooth nuclear envelope (AC-11)  Nuclear: Punctate nuclear envelope (AC-12)  Nuclear: Pleomorphic (AC-13,14)  Nuclear: PCNA-like (AC-13)  Nuclear: CENP-F-like (AC-14) | o  o  o  o  o  o  o  o  o  o  o  o  o  o  o  o  o  o  o | o  o  o  o  o  o  o  o  o  o  o  o  o  o  o  o  o  o  o | o  o  o  o  o  o  o  o  o  o  o  o  o  o  o  o  o  o  o | o  o  o  o  o  o  o  o  o  o  o  o  o  o  o  o  o  o  o | o  o  o  o  o  o  o  o  o  o  o  o  o  o  o  o  o  o  o |
| Cytoplasmic: Fibrillar (AC-15,16,17)  Cytoplasmic: Linear/actin (AC-15)  Cytoplasmic: Filamentous/microtubules (AC-16)  Cytoplasmic: Segmental (AC-17)  Cytoplasmic: Speckled (AC-18,19,20)  Cytoplasmic: Discrete dots (AC-18)  Cytoplasmic: Dense Fine Speckled (AC-19)  Cytoplasmic: Fine speckled (AC-20)  Cytoplasmic: Reticular/AMA (AC-21)  Cytoplasmic: Polar/Golgi like (AC-22)  Cytoplasmic: Rods and rings (AC-23) | o  o  o  o  o  o  o  o  o  o  o | o  o  o  o  o  o  o  o  o  o  o | o  o  o  o  o  o  o  o  o  o  o | o  o  o  o  o  o  o  o  o  o  o | o  o  o  o  o  o  o  o  o  o  o |
| Mitotic: Centrosome (AC-24)  Mitotic: Spindle fibers (AC-25)  Mitotic: NuMa-Like (AC-26)  Mitotic: Intercellular bridge (AC-27)  Mitotic: Mitotic chromosomal envelope (AC-28) | o  o  o  o  o | o  o  o  o  o | o  o  o  o  o | o  o  o  o  o | o  o  o  o  o |

**5. ANA IIF reporting (3)**

5.1 Indicate the patterns you associate with a specific clinical disease and choose

the disease(s) in mind: SLE, SjS, SSc, PM/DM, MCTD, PBC, limited cutaneous

SSc, AIH, RA, other (please specify)

| Nuclear: Homogeneous (AC-1)  Nuclear: Speckled (AC-2,4,5)  Nuclear: Dense fine speckled (AC-2)  Nuclear: Fine Speckled (AC-4)  Nuclear: Large/Coarse speckled (AC-5)  Nuclear: Centromere (AC-3)  Nuclear: Nuclear dots (AC-6,7)  Nuclear: Multiple nuclear dots (AC-6)  Nuclear: Few nuclear dots (AC-7)  Nuclear: Nucleolar (AC-8,9,10)  Nuclear: Homogeneous nucleolar (AC-8)  Nuclear: Clumpy nucleolar (AC-9)  Nuclear: Punctate nucleolar (AC-10)  Nuclear: Nuclear envelope (AC-11,12)  Nuclear: Smooth nuclear envelope (AC-11)  Nuclear: Punctate nuclear envelope (AC-12)  Nuclear: Pleomorphic (AC-13,14)  Nuclear: PCNA-like (AC-13)  Nuclear: CENP-F-like (AC-14) |  |
| --- | --- |
| Cytoplasmic: Fibrillar (AC-15,16,17)  Cytoplasmic: Linear/actin (AC-15)  Cytoplasmic: Filamentous/microtubules (AC-16)  Cytoplasmic: Segmental (AC-17)  Cytoplasmic: Speckled (AC-18,19,20)  Cytoplasmic: Discrete dots (AC-18)  Cytoplasmic: Dense Fine Speckled (AC-19)  Cytoplasmic: Fine speckled (AC-20)  Cytoplasmic: Reticular/AMA (AC-21)  Cytoplasmic: Polar/Golgi like (AC-22)  Cytoplasmic: Rods and rings (AC-23) |  |
| Mitotic: Centrosome (AC-24)  Mitotic: Spindle fibers (AC-25)  Mitotic: NuMa-Like (AC-26)  Mitotic: Intercellular bridge (AC-27)  Mitotic: Mitotic chromosomal envelope (AC-28) |  |

**6. ANA IIF reporting (4)**

6.1 Do you consider cytoplasmic ANA IIF staining as:

Please choose only one of the following:

- ANA positive
- ANA negative
- not applicable

6.2 Do you consider it important that a nucleolar pattern is specified as clumpy, punctate or homogenous?

Please choose only one of the following:

- yes
- no
- not applicable

6.3 Do you consider it important that nuclear dots are specified as multiple or few?

Please choose only one of the following:

- yes
- no
- not applicable

6.4 Do you consider it important that a speckled cytoplasmic staining is specified as discrete dots, dense fine speckled or fine speckled?

Please choose only one of the following:

- yes
- no
- not applicable

6.5 Do you consider it important that a speckled cytoplasmic staining is specified as mitochondrial like?

Please choose only one of the following:

- yes
- no
- not applicable

6.6 Do you consider it important that a fibrillary cytoplasmatic staining is specified as linear, filamentous or segmental? *

Please choose only one of the following:

- yes
- no
- not applicable

6.7 Do you consider it clinically important that ANA IIF titers of nuclear patterns are reported:

Please choose only one of the following:

- quantitative by light intensity score or titer
- semi-quantitative by +, ++, ...
- not at all
- not applicable

6.8 Do you consider it clinically important that ANA IIF titers of cytoplasmatic patterns are reported:

Please choose only one of the following:

- quantitative by light intensity score or titer
- semi-quantitative by +, ++, ...
- not at all
- not applicable

6.9 Do you consider it clinically important that ANA IIF titers of mitotic patterns are reported:

Please choose only one of the following:

- quantitative by light intensity score or titer
- semi-quantitative by +, ++, ...
- not at all
- not applicable

6.10 Do you take medical decisions based on an ANA IIF titer?

Please choose only one of the following:

- no
- yes, please exemplify
- not applicable

Make a comment on your choice here:

6.11 Do you take medical decisions based on specific anti-ENA/dsDNA antibody test results (e.g. anti-SSA, Jo-1)?

Please choose only one of the following:

- no
- yes, please exemplify
- not applicable

Make a comment on your choice here:

6.12 Do you experience differences in results depending on the laboratory?

Please choose only one of the following:

- yes, regarding ANA IIF analysis
- yes, regarding specific anti-ENA/dsDNA antibody tests
- yes, regarding ANA IIF analysis and specific anti-
- ENA/dsDNA antibody tests
- no
- not applicable

We thank you for your participation and will keep you informed on the outcome of the survey.

Submit your survey.
