# Supplementary material for: Current laboratory and clinical practices in reporting and interpreting anti-nuclear antibody indirect immunofluorescence (ANA IIF) patterns: results of an international survey
Source: Auto Immun Highlights. 2020 Nov 23;11(1):17. doi: 10.1186/s13317-020-00139-9 (PMC7684889; doi:10.1186/s13317-020-00139-9)
Supplement: Supplementary file 2 — Additional file 2. International EASI questionnaire ANA IIF patterns for laboratory professionals. [file 13317_2020_139_MOESM2_ESM.docx]

**Supplemental data International EASI questionnaire ANA IIF patterns for laboratory professionals.**

**Accompanying letter**

Dear colleague,

We invite you to participate in a survey on antinuclear antibody (ANA) testing organized by the Belgian branch of the European Autoimmunity Standardization Initiative (EASI) and supported by ICAP.

EASI was founded in 2001 with the intention of improving diagnostics in chronic rheumatic disorders by strengthening the collaboration between clinical and laboratory scientists responsible for autoimmune diagnostics in Europe. One of the aims of EASI is to harmonize algorithms for cost-effective and rational autoantibody testing and the international conceptualization of standardization in this area. In this context, the recent International Consensus on ANA Patterns (ICAP) initiative, in which a standardized way to report ANA patterns is proposed, means an important step in this direction. To extend this initiative and gather worldwide information on ANA pattern recognition and the clinical relevance of specific ANA patterns, we’d like to ask for your cooperation in completing the survey. Two different surveys have been established: one for laboratory professionals performing ANA tests and another for clinicians.

The survey contains 24 questions and takes about 15 minutes to complete. It is possible to save the survey after each page of questions and to continue later. Hereto, click RESUME LATER. The survey will be open till 31/03/2019.

Please have a look at the ICAP website (www.anapatterns.org) before filling out the questionnaire. Your information will be handled confidentially and processed anonymously.

We thank you in advance for your participation.

Sincerely,

In name of the Belgian EASI working group: Apr. Lieve Van Hoovels, Apr. Sofie Schouwers, Prof. Xavier Bossuyt, Dr. Sylvia Broeders;

And of the International Consensus on ANA patters (ICAP) organizers: Dr. Edward Chan, Dr. Luis Andrade

There are 24 questions in this survey

**1. Laboratory setting**

1.1 In which country is your organization located?

Please choose only one of the following: (< List of countries in alphabetic order)

1.2 In which type of organization are your working?

Please choose only one of the following:

- Academic institution
- Clinical/Diagnostic Laboratory
- Hospital/Clinic
- Industry/Business
- Other

1.3 At which date did you consult http://www.anapatterns.org/?

Please enter a data:

1.4 Is your laboratory

Please choose only one of the following:

- primary setting
- secondary setting
- tertiary setting
- not applicable

1.5 Do you consider your laboratory as an expert-level or competent-level (routine) laboratory for indirect immunofluorescence (IIF) on HEp-2 (for definition cfr. HEp-2 ANA IIF patterns classification tree on www.anapatterns.org):

Please choose only one of the following:

- expert-level
- competent-level
- not applicable

**2. ANA IIF reporting**

2.1 Do you report the following patterns?

Please choose the appropriate response for each item:

|  | Yes | No |
| --- | --- | --- |
| Nuclear: Homogeneous (AC-1)  Nuclear: Speckled (AC-2,4,5)  Nuclear: Dense fine speckled (AC-2)  Nuclear: Fine Speckled (AC-4)  Nuclear: Large/Coarse speckled (AC-5)  Nuclear: Centromere (AC-3)  Nuclear: Nuclear dots (AC-6,7)  Nuclear: Multiple nuclear dots (AC-6)  Nuclear: Few nuclear dots (AC-7)  Nuclear: Nucleolar (AC-8,9,10)  Nuclear: Homogeneous nucleolar (AC-8)  Nuclear: Clumpy nucleolar (AC-9)  Nuclear: Punctate nucleolar (AC-10)  Nuclear: Nuclear envelope (AC-11,12)  Nuclear: Smooth nuclear envelope (AC-11)  Nuclear: Punctate nuclear envelope (AC-12)  Nuclear: Pleomorphic (AC-13,14)  Nuclear: PCNA-like (AC-13)  Nuclear: CENP-F-like (AC-14) | o  o  o  o  o  o  o  o  o  o  o  o  o  o  o  o  o  o  o | o  o  o  o  o  o  o  o  o  o  o  o  o  o  o  o  o  o  o |
| Cytoplasmic: Fibrillar (AC-15,16,17)  Cytoplasmic: Linear/actin (AC-15)  Cytoplasmic: Filamentous/microtubules (AC-16)  Cytoplasmic: Segmental (AC-17)  Cytoplasmic: Speckled (AC-18,19,20)  Cytoplasmic: Discrete dots (AC-18)  Cytoplasmic: Dense Fine Speckled (AC-19)  Cytoplasmic: Fine speckled (AC-20)  Cytoplasmic: Reticular/AMA (AC-21)  Cytoplasmic: Polar/Golgi like (AC-22)  Cytoplasmic: Rods and rings (AC-23) | o  o  o  o  o  o  o  o  o  o  o | o  o  o  o  o  o  o  o  o  o  o |
| Mitotic: Centrosome (AC-24)  Mitotic: Spindle fibers (AC-25)  Mitotic: NuMa-Like (AC-26)  Mitotic: Intercellular bridge (AC-27)  Mitotic: Mitotic chromosomal envelope (AC-28) | o  o  o  o  o | o  o  o  o  o |

2.2 Do you agree with the classification (nuclear/cytoplasmic/mitotic) of this pattern?

Please choose the appropriate response for each item:

|  | Yes | No |
| --- | --- | --- |
| Nuclear: Homogeneous (AC-1)  Nuclear: Speckled (AC-2,4,5)  Nuclear: Dense fine speckled (AC-2)  Nuclear: Fine Speckled (AC-4)  Nuclear: Large/Coarse speckled (AC-5)  Nuclear: Centromere (AC-3)  Nuclear: Nuclear dots (AC-6,7)  Nuclear: Multiple nuclear dots (AC-6)  Nuclear: Few nuclear dots (AC-7)  Nuclear: Nucleolar (AC-8,9,10)  Nuclear: Homogeneous nucleolar (AC-8)  Nuclear: Clumpy nucleolar (AC-9)  Nuclear: Punctate nucleolar (AC-10)  Nuclear: Nuclear envelope (AC-11,12)  Nuclear: Smooth nuclear envelope (AC-11)  Nuclear: Punctate nuclear envelope (AC-12)  Nuclear: Pleomorphic (AC-13,14)  Nuclear: PCNA-like (AC-13)  Nuclear: CENP-F-like (AC-14) | o  o  o  o  o  o  o  o  o  o  o  o  o  o  o  o  o  o  o | o  o  o  o  o  o  o  o  o  o  o  o  o  o  o  o  o  o  o |
| Cytoplasmic: Fibrillar (AC-15,16,17)  Cytoplasmic: Linear/actin (AC-15)  Cytoplasmic: Filamentous/microtubules (AC-16)  Cytoplasmic: Segmental (AC-17)  Cytoplasmic: Speckled (AC-18,19,20)  Cytoplasmic: Discrete dots (AC-18)  Cytoplasmic: Dense Fine Speckled (AC-19)  Cytoplasmic: Fine speckled (AC-20)  Cytoplasmic: Reticular/AMA (AC-21)  Cytoplasmic: Polar/Golgi like (AC-22)  Cytoplasmic: Rods and rings (AC-23) | o  o  o  o  o  o  o  o  o  o  o | o  o  o  o  o  o  o  o  o  o  o |
| Mitotic: Centrosome (AC-24)  Mitotic: Spindle fibers (AC-25)  Mitotic: NuMa-Like (AC-26)  Mitotic: Intercellular bridge (AC-27)  Mitotic: Mitotic chromosomal envelope (AC-28) | o  o  o  o  o | o  o  o  o  o |

2.3 Do you consider the pattern competent- or expert-level of reporting?

Please choose the appropriate response for each item:

|  | Competent | Expert |
| --- | --- | --- |
| Nuclear: Homogeneous (AC-1)  Nuclear: Speckled (AC-2,4,5)  Nuclear: Dense fine speckled (AC-2)  Nuclear: Fine Speckled (AC-4)  Nuclear: Large/Coarse speckled (AC-5)  Nuclear: Centromere (AC-3)  Nuclear: Nuclear dots (AC-6,7)  Nuclear: Multiple nuclear dots (AC-6)  Nuclear: Few nuclear dots (AC-7)  Nuclear: Nucleolar (AC-8,9,10)  Nuclear: Homogeneous nucleolar (AC-8)  Nuclear: Clumpy nucleolar (AC-9)  Nuclear: Punctate nucleolar (AC-10)  Nuclear: Nuclear envelope (AC-11,12)  Nuclear: Smooth nuclear envelope (AC-11)  Nuclear: Punctate nuclear envelope (AC-12)  Nuclear: Pleomorphic (AC-13,14)  Nuclear: PCNA-like (AC-13)  Nuclear: CENP-F-like (AC-14) | o  o  o  o  o  o  o  o  o  o  o  o  o  o  o  o  o  o  o | o  o  o  o  o  o  o  o  o  o  o  o  o  o  o  o  o  o  o |
| Cytoplasmic: Fibrillar (AC-15,16,17)  Cytoplasmic: Linear/actin (AC-15)  Cytoplasmic: Filamentous/microtubules (AC-16)  Cytoplasmic: Segmental (AC-17)  Cytoplasmic: Speckled (AC-18,19,20)  Cytoplasmic: Discrete dots (AC-18)  Cytoplasmic: Dense Fine Speckled (AC-19)  Cytoplasmic: Fine speckled (AC-20)  Cytoplasmic: Reticular/AMA (AC-21)  Cytoplasmic: Polar/Golgi like (AC-22)  Cytoplasmic: Rods and rings (AC-23) | o  o  o  o  o  o  o  o  o  o  o | o  o  o  o  o  o  o  o  o  o  o |
| Mitotic: Centrosome (AC-24)  Mitotic: Spindle fibers (AC-25)  Mitotic: NuMa-Like (AC-26)  Mitotic: Intercellular bridge (AC-27)  Mitotic: Mitotic chromosomal envelope (AC-28) | o  o  o  o  o | o  o  o  o  o |

2.4 How do you estimate the clinical relevance of this pattern: 1 (clinically not important) and 5 (clinically very important)?

Please choose the appropriate response for each item:

|  | 1 | 2 | 3 | 4 | 5 |
| --- | --- | --- | --- | --- | --- |
| Nuclear: Homogeneous (AC-1)  Nuclear: Speckled (AC-2,4,5)  Nuclear: Dense fine speckled (AC-2)  Nuclear: Fine Speckled (AC-4)  Nuclear: Large/Coarse speckled (AC-5)  Nuclear: Centromere (AC-3)  Nuclear: Nuclear dots (AC-6,7)  Nuclear: Multiple nuclear dots (AC-6)  Nuclear: Few nuclear dots (AC-7)  Nuclear: Nucleolar (AC-8,9,10)  Nuclear: Homogeneous nucleolar (AC-8)  Nuclear: Clumpy nucleolar (AC-9)  Nuclear: Punctate nucleolar (AC-10)  Nuclear: Nuclear envelope (AC-11,12)  Nuclear: Smooth nuclear envelope (AC-11)  Nuclear: Punctate nuclear envelope (AC-12)  Nuclear: Pleomorphic (AC-13,14)  Nuclear: PCNA-like (AC-13)  Nuclear: CENP-F-like (AC-14) | o  o  o  o  o  o  o  o  o  o  o  o  o  o  o  o  o  o  o | o  o  o  o  o  o  o  o  o  o  o  o  o  o  o  o  o  o  o | o  o  o  o  o  o  o  o  o  o  o  o  o  o  o  o  o  o  o | o  o  o  o  o  o  o  o  o  o  o  o  o  o  o  o  o  o  o | o  o  o  o  o  o  o  o  o  o  o  o  o  o  o  o  o  o  o |
| Cytoplasmic: Fibrillar (AC-15,16,17)  Cytoplasmic: Linear/actin (AC-15)  Cytoplasmic: Filamentous/microtubules (AC-16)  Cytoplasmic: Segmental (AC-17)  Cytoplasmic: Speckled (AC-18,19,20)  Cytoplasmic: Discrete dots (AC-18)  Cytoplasmic: Dense Fine Speckled (AC-19)  Cytoplasmic: Fine speckled (AC-20)  Cytoplasmic: Reticular/AMA (AC-21)  Cytoplasmic: Polar/Golgi like (AC-22)  Cytoplasmic: Rods and rings (AC-23) | o  o  o  o  o  o  o  o  o  o  o | o  o  o  o  o  o  o  o  o  o  o | o  o  o  o  o  o  o  o  o  o  o | o  o  o  o  o  o  o  o  o  o  o | o  o  o  o  o  o  o  o  o  o  o |
| Mitotic: Centrosome (AC-24)  Mitotic: Spindle fibers (AC-25)  Mitotic: NuMa-Like (AC-26)  Mitotic: Intercellular bridge (AC-27)  Mitotic: Mitotic chromosomal envelope (AC-28) | o  o  o  o  o | o  o  o  o  o | o  o  o  o  o | o  o  o  o  o | o  o  o  o  o |

2.5 Are there ANA IIF patterns that you report and that are not indicated in the previous tables?

Please choose only one of the following:

- no
- yes, please specify:

Make a comment on your choice here:

**3. ANA IIF reporting**

3.1 Do you consider cytoplasmic ANA IIF staining as:

Please choose only one of the following:

- ANA positive
- ANA negative
- not applicable

3.2 Do you confirm ANA IIF results with specific ENA/dsDNA antibody tests?

Please choose only one of the following:

- yes, always when ANA IIF is positive
- yes, always when ANA IIF is positive and on clinical indication when ANA IIF is negative
- yes, only when ANA IIF is positive and specific ENA/dsDNA antibody tests are requested
- yes, always when specific ENA/dsDNA antibody tests are requested
- no, in case the ANA pattern indicates an antibody for which a ENA test is not available
- no
- not applicable

3.3 Do you review the reported ANA IIF pattern after specific ENA/dsDNA antibody confirmation tests?

Please choose only one of the following:

- yes
- no
- not applicable

3.4 Do you change the reported ANA IIF pattern according to specific ENA/dsDNA antibody confirmation tests?

Please choose only one of the following:

- yes, please specify
- no
- no, but I add a comment
- not applicable

Make a comment on your choice here:

3.5 What do you consider more clinically important

Please choose only one of the following:

- a specific ANA IIF pattern
- the identification of a specific ENA/dsDNA antibody
- a corresponding ANA IIF pattern and specific ENA/dsDNA antibody identification
- not applicable

3.6 Do you consider it important that a nucleolar pattern is specified as clumpy, punctate or homogenous?

Please choose only one of the following:

- yes
- no
- not applicable

3.7 Do you consider it important that nuclear dots are specified as multiple or few?

Please choose only one of the following:

- yes
- no
- not applicable

3.8 Do you consider it important that a speckled cytoplasmic staining is specified as discrete dots, dense fine speckled or fine speckled?

Please choose only one of the following:

- yes
- no
- not applicable

3.9 Do you consider it important that a speckled cytoplasmic staining is specified as mitochondrial like?

Please choose only one of the following:

- yes
- no
- not applicable

3.10 Do you consider it important that a fibrillary cytoplasmatic staining is specified as linear, filamentous or segmental?

Please choose only one of the following:

- yes
- no
- not applicable

3.11 Do you consider it clinically important that ANA IIF titers of nuclear patterns are reported:

Please choose only one of the following:

- quantitative by light intensity score or titer
- semi-quantitative by +, ++, ...
- not at all
- not applicable

3.12 Do you consider it clinically important that ANA IIF titers of cytoplasmatic patterns are reported:

Please choose only one of the following:

- quantitative by light intensity score or titer
- semi-quantitative by +, ++, ...
- not at all
- not applicable

3.13 Do you consider it clinically important that ANA IIF titers of mitotic patterns are reported:

Please choose only one of the following:

- quantitative by light intensity score or titer
- semi-quantitative by +, ++, ...
- not at all
- not applicable

3.14 Do you automatically report a comment regarding the clinically relevance of the ANA IIF pattern with the results?

Please choose only one of the following:

- yes
- no
- only in specific cases (please specify)
- not applicable

Make a comment on your choice here:

We thank you for your participation and will keep you informed on the outcome of the survey.

Submit your survey.
