# Supplementary material for: Current laboratory and clinical practices in reporting and interpreting anti-nuclear antibody indirect immunofluorescence (ANA IIF) patterns: results of an international survey
Source: Auto Immun Highlights. 2020 Nov 23;11(1):17. doi: 10.1186/s13317-020-00139-9 (PMC7684889; doi:10.1186/s13317-020-00139-9)
Supplement: Supplementary file 3 — Additional file 3. Table S5. Demographic differences in confirming ANA IIF results with specific tests for anti-ENA and anti-dsDNA. [file 13317_2020_139_MOESM3_ESM.docx]

**Supplemental data Table 5.** Demographic differences in confirming ANA IIF results with specific tests for anti-ENA and anti-dsDNA.

The table shows the fraction of laboratories that confirm ANA IIF results with specific ENA/dsDNA antibody tests, and under which condition this is performed (always or only when requested). The results are presented as percentage (%) of the total responders per geographic continent.

| % | Africa  (%)  n=30 | Asia  (%)  n=49 | Australia (%)  n=5 | Europe (%)  n=213 | North  America  (%)  n=30 | South  America  (%)  n=50 |
| --- | --- | --- | --- | --- | --- | --- |
| yes, always when ANA IIF is positive and on clinical indication when ANA IIF is negative | 33.3 | 24.5 | **40.0** | **40.8** | 13.3 | 10.0 |
| Yes, only when ANA IIF is positive and specific ENA/dsDNA antibody tests are requested | 16.7 | 28.6 | 20.0 | 15.5 | 10.0 | 24.0 |
| Yes, always when ANA IIF is positive | 16.7 | 16.3 | 20.0 | 20.7 | 23.3 | 10.0 |
| Yes, always when specific ENA/dsDNA antibody tests are requested | 33.3 | 16.3 | 20.0 | 16.4 | **46.7** | **44.0** |
| no | 0.0 | 10.2 | 0.0 | 4.2 | 6.7 | 10.0 |
| No, in case the ANA pattern indicates an antibody for which an ENA test is not available | 0.0 | 4.1 | 0.0 | 2.3 | 0.0 | 2.0 |
