# Supplementary material for: Current laboratory and clinical practices in reporting and interpreting anti-nuclear antibody indirect immunofluorescence (ANA IIF) patterns: results of an international survey
Source: Auto Immun Highlights. 2020 Nov 23;11(1):17. doi: 10.1186/s13317-020-00139-9 (PMC7684889; doi:10.1186/s13317-020-00139-9)
Supplement: Supplementary file 4 — Additional file 4. Table S6. Overview of the fraction of respondents (laboratory professionals or clinicians) that considered it important to report the fluorescence intensity of nuclear, cytoplasmic or mitotic ANA IIF patterns. [file 13317_2020_139_MOESM4_ESM.docx]

**Supplemental data Table 6.** Overview of the fraction of respondents (laboratory professionals or clinicians) that considered it important to report the fluorescence intensity of nuclear, cytoplasmic or mitotic ANA IIF patterns.

| **ANA IIF pattern** |  | **n** | **Not at all (%)** | **Qualitative by +. ++. … and semi-quantitative (%)** | **Quantitative by light intensity score or titer (%)** |
| --- | --- | --- | --- | --- | --- |
| Nuclear | Lab | 352 | 4.5 | 16.5 | 79.0 |
|  | Clinician | 110 | 8.2 | 18.2 | 73.6 |
| Cytoplasmic | Lab | 346 | 32.9 | 19.4 | 47.7 |
|  | Clinician | 107 | 22.4 | 20.6 | 57.0 |
| Mitotic | Lab | 344 | 43.3 | 14.2 | 42.4 |
|  | Clinician | 104 | 32.7 | 23.1 | 44.2 |
